# Pre-term and post-term births: predictors and implications on neonatal mortality in Northern Ethiopia

**DOI:** 10.1186/s12912-016-0170-6

**Published:** 2016-08-05

**Authors:** Hayelom Gebrekirstos Mengesha, Wondwossen Terefe Lerebo, Abadi Kidanemariam, Gebremedhin Gebrezgiabher, Yemane Berhane

**Affiliations:** 1 College of Medicine and Health Sciences, Adigrat University, Adigirat, Ethiopia; 2 School of Public Health, College of Health Sciences, Mekelle University, Mekelle, Ethiopia; 3 Department of Nursing, Adigrat University, Adigrat, Ethiopia; 4 Department of Midwifery, Adigrat University, Adigrat, Ethiopia; 5 Department of Nursing, Adigrat University, Adigrat, Ethiopia

## Abstract

**Background:**

Pre-term and post-term births are major determinants of neonatal mortality, including short- and long-term morbidity. In developing countries, where pre-term and post-term births are disproportionately common, the magnitude and underlying causes are not well understood, and evidence is required to design appropriate interventions. This study measured the incidence and identified risk factors of pre-term birth and post-term births in Ethiopia. In addition, it examined the effects of pre-term and post-term birth on neonatal mortality.

**Method:**

This study is a portion of prospective cohort study conducted on 1152 live births born between April and July 2014 in seven hospitals in Tigray region, Northern Ethiopia. Neonatal mortality and birth outcomes were considered as dependent variables. Data were collected using a structured questionnaire and weekly neonatal follow up directed at midwives. Data were described using frequency, percentage, ratio of relative risk (RRR), and 95 % confidence interval (CI). We used multinomial and binary logistic regression to identify independent predictors of birth outcome and neonatal mortality respectively.

**Result:**

The prevalence of pre-term and post term births was 8.1 % and 6.0 % respectively. Underweight maternal body mass index (RRR: 0.47, CI: 0.22–0.99), medium reported income (RRR: 0.26, CI: 0.12–0.5), length of neonate (RRR: 0.05, CI: 0.01–0.41), and multiple births (RRR: 2.86, CI: 1.4–5.650) were associated with pre-term birth. Predictors for post-term birth were overweight maternal body mass index (RRR: 3.88, CI: 1.01–15.05), high reported income mothers (RRR: 2.17, CI:1.1–4.3), as well as unmarried, widowed and divorced marital status (RRR:2.43, CI:1.02–5.80). With regards to binary logistic regression, pre-term birth (RR: 2.45, CI: 1.45–4.04) was an independent predictor for neonatal mortality, but this was not true for post-term births (RR: 0.45, CI: 0.07–2.96).

**Conclusion:**

Socioeconomic and proximate factors are important predictors for pre-term and post-term births. Empowering women in terms of income status and controlling body mass index within the normal range are recommended. In addition, early detection and close antenatal follow-ups for mothers, who are at risk before and during pregnancy, are necessary to prevent both pre-term and post-term births.

## Background

Labour outcomes are the essence of successful prenatal programs and the launching of successful post-natal health. Pre-term and post-term births are defined as live births occurring at less than 37 weeks and at 42 or more weeks, respectively, and are associated with both short- and long-term childhood mortality and morbidities [1–5]. In recent studies, the prevalence of pre-term and post-term births are estimated to be 10 % and 5–10 %, respectively [6, 7]. Disparity in the burden of pre-term and post-term births is also widely reported [7, 8]. The highest burden of incidence rates are reported in Sub-Saharan Africa (12.0 %), and Eastern Africa (14.3 %), as compared to Europe (6.2 %) and Asia (9.1 %) [7, 8].

The aetiology of pre-term birth is multi-factorial and it is affected by social, psychological, biological and genetic factors. Its prevalence and risk factors depends on geographic and demographic features of the population studied. Hence, the results of studies from one area might not be applicable to another [6, 9, 10]. In general, previous studies indicated the factors associated with pre-term birth are maternal complications, condition of fetus, genetic influences, environmental exposure, infertility treatments, behavioural and socioeconomic factors, such as income, inadequate prenatal care, and iatrogenic prematurity [6, 11, 12]. Pre-term birth complications account for 35 % of the estimated 3.1 million global neonatal deaths [4], and are the second leading cause of death in children under 5 years of age [6, 9]. Both pre-term and post-term births are associated with poor short and long-term health outcomes. Pre-term infants face higher risk of several disabilities, including neuro-developmental impairments, gastrointestinal complications, cerebral palsy, sensory deficits, learning disabilities, and respiratory illnesses [13].

The morbidities associated with pre-term births often extends to later life resulting in physical, psychological, and economic costs [6, 14]. While there has been significant progress in care for premature infants, reducing the prevalence of post-term birth is more challenging and rates have even increased in some regions [15–17]. Recent studies showed that up to 13 % of post-term newborns develop neurological or developmental disorder by the age of 5 years and disproportionately exhibit attention deficit or hyperactivity-related problem behaviors [1, 18].

Evidence on the effect of post-term birth on neonatal mortality is not consistent. Some studies reported that post-term birth is associated with neonatal death (1, 4), while no association was observed in another study [19]. Post-term pregnancy has been associated with null parity, advanced maternal age and obesity, caesarean section-induced labour, prior post-term pregnancy, male gender of the fetus, and genetic factors in the literature [18, 20, 21].

To our knowledge, in Ethiopia, there is no a systematic evidence respecting the burden and predictors of pre-term, post-term birth and their associations with neonatal mortality. Evidence of the levels of pre-term and post-term births is important to design evidence based health care interventions to reduce the unacceptable prevalence of neonatal and childhood mortality in developing countries. Therefore, the present study aimed to identify the predictors of pre-term birth and post-term birth as well as the relationship of pre-term and post-term birth to neonatal mortality in Northern Ethiopia.

## Methods

### Study design

This report reflects findings from a portion of a large prospective observational study undertaken in one of the nine administrative regions of Ethiopia to investigate pregnancy outcomes and neonatal survival. The current study is a cross-sectional survey and analysis of prospective data on 1162 neonates delivered at selected hospitals between April and July 2014 in Tigray, Ethiopia.

### Study setting and population

This study was part of a study conducted in seven randomly selected public hospitals in Tigray Region, located in northern Ethiopia. The Ethiopian healthcare system has a decentralized four-tier system of primary, district, zonal, and specialized hospitals. The primary care includes rural health posts nested into health centers serving populations of approximately 25,000. District hospitals form the tiers expected to serve catchments of 250,000. Zonal hospitals and specialized hospital serve 500,000 and 5,000,000 people, respectively. In Tigray Region, one of the nine administrative regions and two administrative cities in Ethiopia, there are 15 public hospitals categorized as zonal (6), one specialized hospitals (1), and the district and primary health care (8). All hospitals provide comprehensive maternal and newborn services including delivery service free of charge. Seven hospitals were selected by simple random sampling from across the region using lottery method [22].

### Data collection

Data were collected using a pre-tested structured questionnaire and checklist partially based on the World Health Organization’s verbal autopsy for neonatal deaths [23]. Data on maternal and obstetric characteristics were derived from the antenatal care record, through a post-partum interview with the mother and/or assessment of the neonate and mother. Data collectors interviewed all mothers, within the first 6 h after delivery, who experienced a live birth at the selected hospitals. Data related to the newborns were collected at birth by midwives and, during follow up, by either midwives or health extension workers. Mothers and neonates were followed for 28 days using three data collection methods/alternatives. On the first contact, the data collector visited the neonate daily, while he/she was in the hospital. At birth, socio-demographic and economic, neonatal, maternal and health service related characteristics were recorded, measured, and assessed. The second data collection method occurred after the mother was discharged, with the data collector communicating with the mother every 7 days either using a phone call or by creating a liaison with the health extension workers, inquiring about the neonatal condition and survival in every phone call. The third data collection alternative occurred for mothers who were not at home during the scheduled visit, when a revisit was arranged. When neonatal or maternal death occurred, the date and time of death was recorded and further contact was discontinued with the participant. To ensure the quality of data collected, one day training for the data collectors and health extension workers was provided on how to collect the data and how to follow the mothers. A pre-test was conducted on 5 % of the sample size to improve clarity and modify the data collection instruments.

### Inclusion and exclusion criteria

Mothers with neonates born at the selected facilities between April and July 2014 were eligible for the study. Mothers referred from other health facilities or coming directly from their homes and admitted to one of the seven participating hospitals within six hour of birth were also included. Mothers who came from other neighbouring regions of Ethiopia, who were not able to speak, who had a psychiatric diagnosis, and/or who had no companion were excluded from the study.

### Assessment and definition of outcome and exposure variables

We used previous literature and Mosley and Chen’s conceptual framework [24] with some modifications as a basis for categorization of the independent variables. This analytical framework is used for child health studies and combines sociological and biological explanatory models into a single conceptual framework by sorting independent variables as either distal or proximal determinants, in creating a hierarchy for analysis. The model set up by Mosley and Chen was adapted to fit specific areas, including neonatal health.

This study had two outcome variables that were analyzed independently. The first outcome variable was neonatal mortality, which is death of a live birth baby within 28 days of birth, categorized into binary outcomes for death or survival. The second outcome variable was birth outcome, which is the onset of the labour determined by gestational age of the live birth. This variable was categorized according to World Health Organization’s definitions of pre-term, term births, and post-term births [25]. Pre-term birth is “any birth before 37completed weeks of gestation or fewer than 259 days since the first day of the women’s last menstrual period” [25]. A term birth is a “birth between 37 completed weeks up to 41 completed weeks of gestation” [25]. A post-term is “any birth after 41 completed weeks of gestation or 294 days and above since the first day of the women’s last menstrual period” [25].

Gestational age was estimated based on the last menstrual period. If mothers were not able to recall their last menstrural period, gestational age was estimated using ultrasound methods.

The independent variables were broadly categorized as distal factors, such as socio-economic and demographic factors (i.e., monthly income, marital status, residence); proximal factors (i.e., maternal, neonatal, and health service related); and immediate variables (i.e., maternal complications).

Maternal complications, which were categorizes into ‘Yes’ or ‘No’ responses on assessment, included presence or absence of obstetric haemorrhage, puerperal sepsis, pyrexia, prolonged labour eclampsia and pre-eclampsia, mal-presentation and mal-positioning, premature rupture of membrane, cord prolapse, obstructed labour, cephalo-pelvic disproportion, emergency Caesarean section, and/or retained placenta. In addition, body mass index (BMI) was one of the maternal factors captured and categorized using the standardized categories of underweight (<18.5), normal (18.5–25), and overweight (>25.0). Neonatal factors considered included birth weight (categorized as low, normal, and overweight), sex, and weight for gestational age.

### Data management and analysis

Birth outcomes were analyzed by categorizing into three outcomes variables (i.e., pre-term, term (base) and post-term) considered at the nominal level. Neonatal mortality was fit in a separate model categorized as death (coded “1”) or survival (coded “0”). Potential predictors for pre-term and post-term births were analyzed on the same model based on the assumption of multinomial logistic regression. The predictors were categorized as 1) immediate factors; 2) proximate factors (i.e., maternal, neonatal, and health service factors); and 3) distal factors (i.e., socio-economic and demographics variables). The independent variables considered for neonatal mortality were pre-term and post-term births.

A bivariate logistic regression was performed, and variables with likelihood ratio test *P*-values of less than 0.05 were retained in the multiple regression models. Variables considered in the final model by using the hierarchical method of multiple regression reflected immediate factors (Model 1) followed by proximal factors (Model 2) and, finally, distal factors being added (Model 3) as predictors of pre-term and post-term births. Multinomial and binary logistic regression was used to estimate the relative risk ratios (RRR) and relative risk (RR) as well as corresponding 95 % confidence interval (CI) for the predictors of birth outcomes and neonatal mortality at *P* < 0.05. During model building, cofounders were checked by looking for 15 % change in the regression coefficient. Interaction between predictor variables was tested at a significance level *P* < 0.05 by including an interaction term in the multivariate model. Multicollinearity among independent predictors was tested using Variance Inflation Factor. Data was entered, cleaned, and analyzed using STATA™ Version 11.0 Statistical Package.

## Results

### Response rate and outcome of the follow up

In this study, we found a high response rate of 99.14 % (*n* = 1152). We excluded 10 mothers from the study due to inconsistency and/or inaccuracy of their questionnaires. During follow-up, most mothers (i.e., 996) were followed by phone calls, with 112 mothers met by health extension workers, with 44 discontinuing due to neonatal death prior to discharge, and 60 lost to follow up.

### Socio-economic and demographic characteristics of participants

Over half (57.2 %) of the mothers were between 25–35 year. Regarding education, 275 (23.9 %) of the study participants were unable to read and write (no formal education). Of all mothers, 327 (32.2 %) were in the low monthly income category (i.e., <500 Ethiopian birr). In this study, we found a prevalence of 93 (8.1 %) and 69 (6 %) pre-term and post-term births, respectively. Our participants showed high rates of pre-term and post-term births of 21 (22.5 %) and 18 (27 %) in Adwa and Adigrat hospitals respectively. Mothers in the medium and high income categories had lower risk of pre-term and higher risk of post-term births with relative risk ratio (RRR: 0.36, CI: 0.19–0.68; RRR: 1.93, CI:1.01–3.71) respectively. Mothers, who were unmarried, widowed, and divorced [81 (7 %)], were at higher risk of post-term births (RRR: 2.58, CI: 1.25–5.29) (Table 1).

**Table 1:** Association of selected socio-demographic characteristics with preterm and post term births on neonates delivered in Tigray, northern Ethiopia, April-July, 2014

Characteristics	Total live birth		Preterm birth	Post term birth	preterm & post term
	No	%	No (RRR^a^, CI)	No (RRR^b^,CI)	*P*-value
Hospital					
Ayder Referral	139	12.1	13	4	
Adwa	198	17.2	21	14	
Lemlem Karl	147	12.8	4	12	
Suhl	143	12.4	15	14	
Kahsay Abera	102	8.8	12	0	
Kidist Mariam	213	18.5	17	7	
Adigrat	210	18.2	11	18	
Residence					
Rural	397	34.5	40 (ref)	21 (ref)	
Urban	755	65.5	53 (0.68, 0.44–1.04)	48 (1.17, 0.69–1.99)	0.08,0.55
Educational status					
Unable to read	275	23.9	34 (ref)	14 (ref)	
Primary	338	29.3	25 (0.56, 0.32–0.97)	19 (1.04, 0.51–2.13)	0.042, 0.89
Secondary	361	31.3	20 (0.41, 0.23–0.7)	18 (0.90, 0.44–1.85)	0.003, 0.78
Tertiary	178	15.5	14 (0.64, 0.33–1.23)	18 (1.99, 0.96–4.14)	0.18, 0.06
Monthly income^b^					
Poor	327	32.2	39 (ref)	14 (ref)	ref
Medium	316	31.1	15 (0.36, 0.19–0.68)	16 (1.09, 0.52–2.29)	0.002,0.80
Rich	373	36.7	29 (0.65, 0.39–1.08)	31 (1.93, 1.01–3.71)	0.097,0.04
Marital status					
Married	1071	93.0	83 (ref)	59 (ref)	ref
Other	81	7.0	10 (1.83,0.9–3.71)	10 (2.58,1.25–5.29)	0.092,0.01

^a^ *RRR* relative risk ratio, *CI* confidence interval, *Ref* reference,^b^Rich = ≥1500 Ethiopian birr (ETB), Medium: 600–1500 ETB, Poor = <600 ETB

### Neonatal and maternal characteristics

Mothers, who had maternal complications during delivery [221 (19.2 %)], showed higher risk of pre-term delivery (RRR: 1.34, CI: 0.67–2.68) over term births. In relation to maternal BMI, 119 (10.6 %) were in the underweight and 96 (8.6 %) overweight categories; however, overweight mothers were found to be at greater risk for a post-term birth (RRR: 3.55, CI: 1.08–11.5) (Table 2).

**Table 2:** Association of selected Maternal and Neonatal characteristics with preterm and post term births on neonates delivered in Tigray, northern Ethiopia, April-July, 2014

Characteristics	Total live births		Preterm birth	Post term birth	Preterm & post term	
	No	%	No (RRR, CI)	No (RRR,CI)	*P*-value	
Current age of mother						
<20 year	95	8.2	10 (ref)	6 (ref)	ref	
20–24 year	328	28.5	24 (0.67, 0.30–1.46)	22 (1.02, 0.4–2.6)	0.672,0.95	
25–35 year	590	57.2	45 (0.68, 0.33–1.42)	29 (0.73, 0.29–1.83)	0.68, 0.51	
≥ 35 year	139	12.1	14 (0.97, 0.41–2.31)	12 (1.39, 0.50–3.88)	0.97, 0.52	
Birth interval						
Not applicable	537	46.6	40 (ref)	34 (ref)		
<Two years	127	11.0	10 (1.08, 0.52–2.32)	10 (1.27, 0.60–2.65)	0.83, 0.52	
>Two years	488	42.4	43 (1.18, 0.75–1.85)	25 (0.81,6.47–1.38)	0.44,0.44	
Ever use of FP						
Yes	650	56.4	48 (ref)	38 (ref)		
No	502	43.6	45 (1.24, 0.82–1.89)	31 (1.08,0.66–1.76)	0.32,0.75	
Disease						
Yes	100	8.7	7 (ref)	5 (ref)		
No	1052	91.3	86 (1.19, 0.53–2.66)	64 (1.24,0.48–3.18)	0,66,0.642	
Maternal complications						
Yes	221	19.2	27	10		
No	930	80.8	66 (0.55, 0.34–0.9)	59 (1.34,0.67–2.68)	0.016,0.395	
Mode of delivery						
Vaginal	846	73.5	75	46		
Caesarean section	241	20.9	13 (0.59, 0.32–1.08)	16 (1.18, 0.65–2.14)	0.01,0.56	
Instrumental	65	5.6	5 (0.91, 0.35–2.35)	7 (2.08, 0.89–4.83)	0.81,0.08	
Sex						
Male	610	52.9	50 (ref)	43 (ref)	ref	
Female	542	47.1	43 (0.55, 0.34–0.89)	35 (1.34, 0.67–2.68)	0.016, 0.39	
Neonatal length						
<46 cm	151	13.4	46 (ref)	3 (ref)		
46–56 cm	914	81.2	45 (0.13, 0.08–0.20)	60 (2.66, 0.82–8.66)	<0.001,0.10	
>56 cm	61	5.4	2 (0.04, 0.05–0.32)	6 (4, 0.96–16.6)	0.002,0.06	
Birth weight						
<2500 gm	121	10.5	50 (111, 15–82)	1 (0.12, 0.016–0.94)	<0.001,004	
2500–3500 gm	853	74.2	42 (8.6, 1.17–63)	50 (0.05–1.00)	0.034,0.05	
>3500 gm	175	15.3	1 (ref)	18 (ref)	ref	
Weight for gestational age						
Appropriate	846	73.5	52 (ref)	47 (ref)		
SGA	125	10.9	17 (2.44, 1.35–4.38)	8 (1.27, 0.58–2.76)	0.003,0.54	
LGA	180	15.6	24 (2.42, 1.44–4.06)	14 (0.15, 0.8–3.0)	0.004,0.26	<0.01,0.158
Birth type						
Single	1057	91.8	70 (ref)	66 (ref)	ref	
Multiple	95	8.2	23 (4.38, 2.57–7.45)	3 (0.60, 0.185–1.97)	<0.001,0.40	
Body mass index						
Underweight	119	10.6	13 (ref)	4 (ref)	ref	
Normal	907	80.8	72 (0.72, 0.38–1.34)	52 (1.69, 0.59–4.78)	0.30,0.32	
overweight	96	8.6	6 (0.59, 0.21–1.61)	11 (3.55, 1.08–11.5)	0.31,0.04	
Hypothermic						
Yes	61	5.3	23 (ref)	6 (ref)	ref	
No	1091	94.7	70 (0.1, 0.05–0.18)	63 (0.35, 0.14–0.86)	<0.001, 0.02	

### Predictors of pre-term and post-term birth

On multiple multivariable analyses, using hierarchical modeling of variables, we found BMI of the mother, birth type (i.e., single versus multiple) of neonate, length of neonate, monthly income, and marital status were independently associated with pre-term and post-term births relative to term births.

The risk for pre-term births relative to term births in a multiple birth scenario (i.e., twins and triplets) was 2.86 times greater relative to singleton births (RRR: 2.86, CI: 1.45–5.65); however, post-term birth was not associated with multiple births given the other factors in the model are held constant. Body length of neonates was also associated with pre-term infants, but not with post-term. Neonates, who were 46–56 cm and ≥56 cm at birth, were found to be 88 % (RRR: 0.12, CI: 0.07–0.21) and 99.95 % (RRR: 0.05, CI: 0.01–0.4) at lower risk of pre-term birth relative to term births. Post-term birth was not associated with length of the neonate. Relative to term births, overweight status was significantly associated with post-term births at a 3.88 times higher relative risk (RRR: 3.88, CI: 1.01–15.05). Underweight maternal status relative to normal weight status showed a decrease in relative risk by a factor of 0.47 of delivering pre-term births relative to term births when other factors are held constant (RRR: 0.47, CI: 0.22–0.99). Marital status was also associated with post-term births. Neonates from mothers who were either widowed, divorced, separated, or unmarried relative to married mothers were 2.43 times at higher relative risk for post-term birth than term births (RRR: 2.43,CI:1.02–5.80), but pre-term births were not significantly associated on the final models. Regarding income status, mothers in the medium income category were 74 % less relative risk to experience a pre-term birth than mothers in the low income category (RRR: 0.26, CI: 0.12–0.50). Mothers in the high income category were 2.17 times more at relative risk for delivering a post-term birth relative with mothers in the low income category (RRR:2.17, CI:1.1–4.3) (Table 3).

**Table 3:** Multivariable regression of the predictors of preterm and post term births delivered in Tigray, Northern Ethiopia, April-July 2014

	Model 1:		Model 2:		Model 3:	
	Immediate	Factors	Proximal	Factors	Distal	Factors
Predictors	RRR& CI preterm	RRR & CI post term	RRR& CI preterm	RRR& CI post term	RRR & CI preterm	RRR & CI post term
Body mass index						
Normal (ref)			ref	ref	ref	ref
Underweight			0.59,0.29–1.2	1.7,0.59–4.82	0.47,0.2–0.99	2.18,0.6–7.2
Overweight			0.57,0.1–1.69	3.34,1.1–11.2	0.5,0.15–1.63	3.88,1.01–15.05
Weight for age						
Normal			ref	ref	ref	ref
SGA			1.03,0.51–2.0	1.66,0.71–3.9	1.02,0.48–2.1	1.56,0.6–3.9
LGA			2.48,1.37–4.4	1.47,0.77–2.8	1.87,0.95–3.6	1.57,0.8–3.0
Multi Birth (“sin”ref)			2.74,1.44–5.2	0.65,0.2–2.31	2.86,1.4–5.65	0.75,0.2–2.7
Female (“male” ref)			1.09,0.67–1.7	1.2,0.76–2.09	1.1,0.65–1.84	1.3,0.77–2.2
Complication (“yes”ref)	0.5,0.34–0.9	1.34,0.7–2.1	0.69,0.4–1.17	1.33,0.66–2.7	0.85,0.47–1.5	1.34,0.6–2.8
Length of neonate						
< 46 cm			ref	ref	ref	ref
46–56 cm			0.15,0.09–0.2	2.57,0.76–8.6	0.12,0.07–0.2	2.3,0.69–3.0
> 56 cm			0.05,0.01–0.4	3.68,0.8–15.8	0.05,0.01–0.4	2.6,0.47–14.3
Monthly Income						
Poor					ref	ref
Medium					0.26,0.12–0.5	1.2,0.55–2.6
Rich					0.6,0.34–1.06	2.17,1.1–4.3
Marital status						
Married					ref	ref
Others					1.62, 0.6–4.2	2.4,1.02–5.8

*BF breast feeding, *ref* reference, *sin* single birth

### Effect of pre-term and post-term births on neonatal mortality

There were 68 neonatal deaths yielding a neonatal mortality rate of 62.5 per 1000 live births. Of these, 42 (62 %) were pre-term and only one was post-term birth. On bivariate analysis, pre-natal birth (RR: 7.06, CI: 4.5–10.9), history of abortion, history of stillbirth, low birth weight, and being a primiparae were associated with high risk of neonatal mortality (Table 4).

**Table 4:** Characteristics of mothers and neonates with neonatal mortality on neonates delivered in Tigray, northern Ethiopia

	Total live births		Neonatal death		
Characteristics	No	%	No	(RR,CI)	*P*-value
Residence					
Rural	397	34.46	38	ref	ref
Urban	755	65.54	30	0.39, 0.24–0.6	<0.001
Preterm					
Yes	93	8.07	42	7.06, 4.5–10.9	<0.001
No	1059	91.93	26	ref	
Post term					
Yes	69	94.01	1	0.23, 0.03–1.6	0.14
No	1083	5.99	67	ref	
Term					
Yes	990	85.94	41	0.24,0.15–0.4	<0.001
No	162	14.06	27	ref	
Neonate out born					
Yes	1085	94.18	18	ref	ref
No	67	5.82	50	5.68, 3.53–9.1	<0.001
History of abortion					
Yes	172	14.93	18	0.49, 0.29–0.8	
No	980	85.07	50	ref	0.007
History of still birth					
Yes	91	7.9	15	ref	
No	1061	92.1	53	0.29, 0.17–0.5	0.006
Birth weight					
Low birth weight	121	10.53	35	10.5, 4.2–25.9	<0.001
Normal	853	74.24	28	1.19, 0.46–3.0	0.715
Overweight	175	15.23	5	ref	ref
Number of children					
Primiparae	610	52.95	35	ref	ref
2–4 children	424	36.81	17	0.7, 0.40–1.23	0.206
Multiple (≥5)	118	10.24	15	2.36, 1.35–4.1	0.022

On multivariable regression of the primary independent variables and other secondary variables using stepwise backward elimination, we found pre-term birth as a significant predictor of neonatal mortality, but post-term births were not significantly associated with neonatal mortality. Pre-term births were at a 2.45 times higher risk for neonatal mortality compared to non-pre-term births (RR: 2.45, CI: 1.45–4.04). However, post-term births were not found to be associated with neonatal mortality (RR: 0.45, CI: 0.07–2.96) (Table 5).

**Table 5:** Multivariable analysis on the association of preterm and post term birth on neonatal mortality in Tigray, northern Ethiopia

Characteristics	(RR,CI)	*P*-value	(ARR^a^,CI)	*p*-value
Residence				
Rural	ref	ref		
Urban	0.39, 0.24–0.62	<0.001		
Preterm				
Yes	7.06, 4.55–10.94	<0.001	2.45, 1.45–4.04	<0.001
No	ref			
Post term				
Yes	0.23, 0.03–1.64	0.14	0.45, 0.07–2.96	0.412
No	ref			
Term				
Yes	0.24, 0.15–0.39	<0.001		
No	ref			
Neonate out				
Yes	ref	ref		
No	5.68,3.53–9.16	<0.001		
History of abortion				
Yes	ref			
No	0.49, 0.29–0.81	0.007	0.47, 0.33–0.67	<0.001
History of still birth				
Yes	ref			
No	0.29, 0.17–0.49	0.006	0.38, 0.29–0.51	<0.001
Birth weight				
Low birth	10.5,4.24–25.9	<0.001	5.17,1.94–13.78	<0.001
Normal	1.19,0.46–3.04	0.715	1.09,0.43–2.80	0.844
Overweight	ref	ref		
Number of children				
Primiparae	ref	ref		
2–4 children	0.7,0.40–1.23	0.206	0.81,0.82–0.82	<0.001
Multiple (≥5)	2.36,1.35–4.12	0.022	1.92,1.48–2.62	<0.001

^a^ *ARR* Adjusted Relative Risk

No confounding, interaction and multicollinearity were detected in this study.

## Discussion

In this study, we determined the predictors of pre-term and post-term births. The overall prevalence of pre-term and post-term births was 8.1 % and 6 % respectively. In addition, we examined the effect of pre-term and post-term births on neonatal mortality. Hence, delivering multiple births, underweight and overweight mothers, short length of the neonate, low and high reported income of mothers, and mothers who were single were independently associated with pre-term and post-term births. The prevalence of pre-term birth was higher than previous reports from Iran and southern Brazil [10, 26] but lower than recent reports from a systematic review of the World Health Organization studies in Bangladesh, Africa, and Debremarkos Ethiopian studies [6, 8, 27]. This variability may be explained in terms of geographical and demographic features of the populations being studied, as pre-term births are known to vary between regions and different populations [6, 9]. In addition, the differences may be attributed to timing of the studies.

Through efforts such as the Millennium Development Goals and health sector development programs, services provided for mothers for reducing and/or preventing pre-term births have been strengthened [16, 17], including within our study area. Thus, these efforts may have contributed to lowering numbers of pre-term births. Similarly, the increasing coverage and accessibility to primary health care services might also have contributed to reducing the prevalence of pre-term births [22].

In the present study, pre-term births were identified based on the last menstrual period, which is reported to underestimate pre-term births [28]. As for the higher rate, our study was conducted only in hospitals where mothers were diagnosed with possible complications. For this reason, they would be more likely to experience pre-term births. However, educated and more affluent mothers may visit specialized hospitals, thus lowering the pre-term birth rate since increased education and higher income are known to lower pre-term birth [29].

The prevalence for post-term birth in our study is lower than findings in two recent studies in Sweden and Holland; however, it falls within the global prevalence range of 5–10 % [6, 7, 18]. The relatively low rate of post-term births may be due to the relatively young age cohort (below 35 years) of the mothers involved in our study. Advanced age is a well-established risk factor for post-term birth [18, 20]. This variance may also relate to inaccurate measurement of the last menstrual period, since some mothers may not able recall their date accurately, may have menstrual irregularities and abortion. This inaccuracy of gestational age could underestimate the post-term birth prevalence in our study.

Multiple birth was an independent predictor for pre-term birth which is consistent with previous findings [30, 31]. Multiple pregnancies are more likely to encounter complications and health risks than their counterparts. As a result, this complication may lead to earlier delivery in their pregnancy either electively or spontaneously. Furthermore, singleton pregnancies are less likely to experience malnutrition than multiple pregnancies [12]. Thus, the nutrition of the mother and neonate may contribute to immaturity of neonate and premature labour. In the present study, neonates from mothers with low reported income were more likely to experience pre-term deliveries. This finding is similar with previous reports in Ethiopia, and Iraq [8, 11, 12]. Socioeconomic factors were another independent predictors for pre-term births [6]. The reason could be low socio-economic status mothers are more vulnerable to low health service utilization, stressful life circumstances, as well as physical and psychological harmful activities, which, in turn, may result in undernutrition and spontaneous pre-term birth.

Conversely, high reported income was significantly associated with post-term birth. This suggests that these mothers may engage in a sedentary lifestyle which may contribute to inappropriate weight gain and associated chronic diseases such as hypertension and diabetes mellitus during pregnancy, known risk factors for post-term birth [32, 33]. We are not able to determine the association between these diseases and post-term birth in our study because of the small sample size and inadequate power to detect the effect. Furthermore, income level was not determined using a wealth indices with appropriate statistical analysis, but rather it was determined based on the response of mothers which may impact on the estimation accuracy.

Underweight mothers were at lower risk to give pre-term birth relative to term birth. Different studies conducted with different study designs and sample sizes indicate maternal underweight or low BMI was associated with pre-term delivery [34–37]. It is expected that low BMI mothers may be exposed to under-nutrition and poor weight gain during pregnancy, resulting in subsequent premature delivery. However, this study finding contradicts with these previous findings. This suggests that the reasons for prematurity were not caused by BMI but there may be other unexplored factors in our study area. But the statistical analysis we used in this study may not robust to declare statistical significance. Hence it needs future study to assess effect of maternal BMI on birth outcome.

Being overweight was associated with post-term births in our study. This finding is in line with previous reports on association of BMI and post-term births in which they reported, as underweight is less associated with post-term births whereas obese and overweight mothers were being associated with post-term births [36, 37]. This might be related to overweight mothers being more susceptible to gestational diabetes mellitus and gestational hypertension/preeclampsia [32, 33], which are known risk factors for post-term births.

Mothers, who were unmarried, widowed, separated and divorced (single), were at higher risks of post-term births, which is inconsistent with previous studies. This might be because unmarried women could be more vulnerable to stressful life, heavy and risky activities, inappropriate weight gain and associated chronic disease; consequently, this may result in post term births. Hence, our study result may give insight for future research in determining the association of marital status with post-term birth.

Our study found that, in our population, 38.23 % of neonatal deaths occurred because of pre-term births. Similarly, previous reports showed that 35 % of all neonatal mortality and 28 % of early neonatal mortality were related to pre-term births [9, 38]. It is apparent that premature neonates are more susceptible to death because they are less adaptive to the external environment and they have immature body systems. Conversely, in our study, post-term birth was not associated with neonatal mortality, although this differed from some previous studies [1, 4]. This may be due to the estimation of gestational age using the last menstrual period, as some authors have suggested there is an overestimation of the post-term births using this recall indicator [28, 39] resulting in term births erroneously being categorized as post-term births.

After adjusting for potential confounders, pre-term births were associated with neonatal mortality but post-term births was not associated with neonatal mortality, which is congruent with previous studies [3, 5].

The strength of this study is the use of a prospective cohort study which shows good validity and reliability of data. We were able to consider a large sample size which is good to detect effect on the predictors.

One limitation of this study is that reliance on the gestational age determined using last menstrual period is known to overestimate or underestimate pre-term and post-term births compared to ultrasound [28, 39]. Another limitation of this study is that it was only conducted in government hospitals; hence, it cannot be generalized to all births as it excludes births in private institutions and home deliveries that will exhibit differing health care intervention levels. We had a 5.2 % lost to follow up which might have resulted in an under- or over-estimation of neonatal mortality. The data were not also primarily collected for studying neonatal mortality, and thus we may miss additional variables that could have an association with birth outcome.

## Conclusion

Overall, this study reported that the prevalence of pre-term and post-term births to be 8 % and 6 % respectively. Low reported income, underweight, multiple birth, and short body length of the neonate were found to be associated with pre-term births, while high reported income, overweight, and being a single mother were associated with post-term births. The study also reports that pre-term births significantly increase the risk of neonatal deaths.

Considering that the global incidences of pre-term birth and its association with neonatal mortality, the World Health Organization recommends a 50 % reduction in pre-term births in developing countries like Ethiopia [40]. Therefore, to achieve this goal, improving and balancing nutritional and socio-economic statuses of mothers is necessary to reduce pre-term and post term births and mitigate their long-term consequences.

This study suggested that focusing on pre-term births rather than on post-term births is necessary to reduce neonatal mortality. Tigray Region Health Bureau, Ethiopia Ministry of Health, Ethiopian policy makers, program planners and other stakeholders who are engaged on improving neonatal health, including pre-term and post-term births, should be made aware of the predictors in order to reduce neonatal mortality and adverse birth outcomes.

## References

[1] ElMarroun H, Zeegers M, Steegers EAP, Schenk JJ, Hofman A, Jaddoe VWV (2012). Post-term birth and the risk of behavioural and emotional problems in early childhood. Int J Epidemiol.

[2] Beltrand J, Soboleva TK, Shorten PR, Derraik JG (2012). Post-term birth is associated with greater risk of obesityin adolescent males. J Pediatr US.

[3] Moster D, Wilcox AJ, Vollset SE, Markestad T, Lie RT. Cerebral palsy among term and postterm births. Jama. 2010;304(9):976–82 10.1001/jama.2010.1271 PMC3711561 20810375

[4] Clausson B, Cnattingius S, Axelsson O (1999). Outcomes of post-term births: the role of fetal growth restriction and malformations. Obstet Gynecol.

[5] Saigal S, Doyle LW (2008). Pre-term birth: an overview of mortality and squeal of pre-term birth from infancy to adulthood. Lancet.

[6] Beck S, Wojdyla D, Say L, Bertrand AP, Merialdi M, Requejo JH (2010). The worldwide incidence of pre-term birth: a systematic review of maternal mortality and morbidity. Bull World Health Organ..

[7] Zeitlin J, Blondel B, Alexander S, Breart G, PERISTAT Group (2007). Variation in rates of post term birth in Europe: reality or artefact?. BJOG.

[8] Bekele T, Amanon A, Gebreslasi KZ (2015). Pre-term birth and associated factors among mothers who gave birth in Debremarkos town health institutions, 2013 institutional based cross sectional study. Gynecol Obstet.

[9] Renzo GCD, Giardina I, Rosati A, Clerici G, Torricelli M, Petraglia F (2011). Maternal risk factors for pre-term birth: a country-based population analysis. Eur J Obstet Gynecol..

[10] Miranda AE, Pinto VM, Szwarcwald CL, Golub ET (2012). Prevalence and correlates of pre-term labor among young parturient women attending public hospitals in Brazil. Rev Panam Salud Publica.

[11] Almeida AC, Jesus AC, Lima PF, Araújo MF, Araújo TM (2012). Maternal risk factors for premature births in a public maternity hospital in Imperatriz-MA. Rev Gaucha Enferm.

[12] Bryan E (2003). The impact of multiple pre-term births on the family. BJOG.

[13] Modarres SZ, Amooian B, Movahed SB, Mohamadi M (2007). Periodontal health in mothers of pre-term and term infants. Taiwan J Obstet Gynecol.

[14] Goldenberg RL, Culhane JF, Iams JD, Rometo R (2008). Epidemiology and cause of pre-term birth. Lancet..

[15] Pennell CE, Jacobsson B, Williams SM, Buus RM (2007). Genetic epidemiologic studies of pre-term birth: guidelines for research. Am J Obstet Gynecol.

[16] Simmons LE, Rubens CE, Darmstadt GL, Gravett MG (2010). Preventing pre-term birth and neonatal mortality: exploring the epidemiology, causes, and interventions. Semin Perinatol.

[17] Morin M, Arnaud C, Germany L, Vayssiere C (2012). Pre-term birth: evolution1994 to 2006. Gynecol Obstet Fertil.

[18] Roos N, Sahlin L, Ekman-Ordeberg G, Kieler H (2010). Maternal risk factors for post-term pregnancy and caesarean delivery following labor induction. Acta Obstetriciaet Gynecologica..

[19] Divon MY, Haglund B, Nisell H, Otterblad PO, Westgren MF (1998). Neonatal mortality in the post-term pregnancy: the impact of gestational age and fetal growth restriction. Am J Obstet Gynecol.

[20] Caughey AB, Snegovskikh VV, Norwitz ER (2008). Post term pregnancy: how can we improve outcomes?. Obstet Gynecol Surv.

[21] Norwitz ER, Snegovskikh VV, Caughey AB (2007). Prolonged pregnancy: when should we intervene?. Clin Obstet Gynecol.

[22] Government of the National State of Tigri (2014). Report on the health status of Tigray region.

[23] World Health Organization (2003). Standard neonatal verbal autopsy questionnaire.

[24] Mosley WH, Chen LC (1984). An analytical framework for the study of child survival in developing countries. Popul Dev Rev.

[25] Liu L, Johnson HL, Cousens S, Perin J, Scott S, Lawn JE (2012). Child Health Epidemiology Reference Group of WHO and UNICEF: Global, regional, and national causes of child Mortality: an updated systematic analysis for 2010 with time trends since 2000. Lancet.

[26] Rahele A, Sadegh H, Mehrdad M, Farhad P, Peymaneh AH (2014). Prevalence and risk factors associated with pre-term birth in Ardabil, Iran. Iran J Reprod Med.

[27] Shah R, Mullany LC, Darmstadt GL, Mannan I, Rahman SM (2014). Incidence and risk factors of pre-term birth in a rural Bangladesh cohort. BMC Pediatr.

[28] Lynch CD, Zhang J (2007). The research implications of the selection of a gestational age estimation method. Paediatr Perinat Epidemiol.

[29] Foster HW, Wu L, Bracken MB, Semenya K, Thomas J (2000). Intergenerational effect of high socioeconomic status on low birth weight and preterm birth in African Americans. Natl Med Assoc.

[30] Al-Dabbagh SA, Al-Taee WY (2006). Risk factors for pre-term birth in Iraq: a case-control study. BMC Pregnancy Childbirth.

[31] Kurdi AM, Mesleh RA, Al-Hakeem MM, Khashoggi TY, Khalifa HM (2004). Multiple pregnancy and pre-term birth. Saudi Med J.

[32] Torloni MR, Bertrand AP, Horta BL (2009). Pre-pregnancy BMI and the risk of gestational diabetes: a systematic review of the literature with meta-analysis. Obstet Rev.

[33] Sohlberg S, Stephansson O, Cnattingius S (2012). Maternal body mass index, height, and risks of preeclampsia. Am J Hypertens.

[34] Parker MG, Ouyang F, Pearson C, Gillman MW, Belfort MB, Hong X (2014). Prepregnancy body mass index and risk of pre-term birth: association heterogeneity by pre-term subgroups. BMC Pregnancy Childbirth.

[35] Al-Salami KS, Alyasin ZT, Hussain RN (2009). Influence of body mass index on pre-term labour. Basrah J Surg.

[36] Halloran DR, Cheng YW, Wall TC, Macones GA, Caughey AB (2012). Effect of maternal weight on post-term delivery. J Perinatol.

[37] Kandeel MS, Sanad ZF, Sayyed TM, Elmenawy SGAE (2014). The effect of body mass index on cervical characteristics and on the length of gestation in low-risk pregnancies. Menoufia Med J.

[38] Lawn JE, Wilczynska-Ketende K, Cousens SN (2006). Estimating the causes of 4 million neonatal deaths in the year 2000. Int J Epidemiol.

[39] Joseph KS, Huang L, Liu S, Ananth CV, Allen AC, Sauve R (2006). Reconciling the high rates of pre-term and post-term birth in the United States. Obstet Gynecol.

[40] Howson C, Kinney M, Lawn J, World Health Organization, March of Dimes, PMNCH, Save the Children (2012). Borntoosoon. The global action report on pre-term birth.

